# Comparative Transcriptome Analysis Between Resistant and Susceptible Rice Cultivars Responding to Striped Stem Borer (SSB), *Chilo suppressalis* (Walker) Infestation

**DOI:** 10.3389/fphys.2018.01717

**Published:** 2018-11-30

**Authors:** Yue Wang, Di Ju, Xueqing Yang, Dianrong Ma, Xiaoqi Wang

**Affiliations:** ^1^Key Laboratory of Economical and Applied Entomology of Liaoning Province, College of Plant Protection, Shenyang Agricultural University, Shenyang, China; ^2^Rice Research Institute, Shenyang Agricultural University, Shenyang, China; ^3^Key Laboratory of Northeast Rice Biology and Breeding, Ministry of Agriculture, Shenyang, China; ^4^Key Laboratory of Northern Japonica Super Rice Breeding, Ministry of Education, Shenyang, China

**Keywords:** *Chilo suppressalis* (Walker), rice, resistance identification, RNA-Seq, systemic induced defense

## Abstract

The striped stem borer, *Chilo suppressalis* (Walker), is a notorious pest of rice that causes large losses in China. Breeding and screening of resistance rice cultivars are effective strategies for *C. suppressalis* management. In this study, insect-resistant traits of 47 rice cultivars were investigated by *C. suppressalis* artificial infestation (AI) both in field and greenhouse experiments, using the susceptible (S) cultivar 1665 as a control. Results suggest that two rice cultivars, namely 1688 and 1654, are resistant (R) and moderately resistant (MR) to *C. suppressalis*, respectively. Then, a comparative transcriptome (RNA-Seq) was *de novo* assembled and differentially expressed genes (DEGs) with altered expression levels were investigated among cultivars 1688, 1654, and 1665, with or without *C. suppressalis* infestation for 24 h. A total of 2569 and 1861 genes were up-regulated, and 3852 and 1861 genes were down-regulated in cultivars 1688 and 1654, respectively after artificial infestation with *C. suppressalis* compared to the non-infested control (CK). For the susceptible cultivar 1665, a total of 882 genes were up-regulated and 3863 genes were down-regulated after artificial infestation with *C. suppressalis* compared to the CK. Twenty four DEGs belong to proteinase inhibitor, lectin and chitinase gene families; plant hormone signal transduction and plant-pathogen interaction pathways were selected as candidate genes to test their possible role in *C. suppressalis* resistance. RT-qPCR results revealed that 13 genes were significantly up-regulated and 8 were significantly down-regulated in the resistant cultivar 1688 with *C. suppressalis* artificial infestation (1688AI) compared to the CK. Three genes, *LTPL164*, *LTPL151*, and *LOC Os11g32100*, showed more than a 10-fold higher expression in 1688AI than in 1688CK, suggesting their potential role in insect resistance. Overall, our results provide an important foundation for further understanding the insect resistance mechanisms of selected resistant varieties that will help us to breed *C. suppressalis* resistant rice varieties.

## Introduction

Rice (*Oryza sativa* L.) is the most widely consumed food crop in the world (Chen et al., 2011), being the food staple for over 1 billion people in China and 2 billion people in other countries (Herdt, 1991). However, rice is frequently attacked by rice stem borers, a major group of lepidopteran pests of rice (Lu et al., 2017) causing annual losses of US$1.69 billion (Sheng et al., 2003). Among these stem borers, *Chilo suppressalis* (Walker), commonly known as the striped stem borer, is a widely distributed destructive pest of rice that has greatly reduced rice production in China (Qu et al., 2003). Rice plant damage by *C. suppressalis* larvae includes boring into the stem and feeding inside, resulting in “dead hearts” (yellowing and withering of the stem) at the vegetative stage and “white heads” at the reproduction stage (Pathak, 1968). Once infested, such a rice plant will fail to produce an effective panicle.

Various control strategies including mechanical, biological, chemical, and cultural control have been used for striped stem borer management (Li, 1982). However, effective management of striped stem borer mainly depends on chemical insecticides (Su et al., 2014). Misuse of insecticides has resulted in severe insect outbreaks, environmental pollution, insecticide resistance, and food security problems (Chen et al., 2011). Thus, newer and safer pest management strategies are urgently needed for rice production in China. Agricultural biotechnology has been extensively explored to solve these problems in China, and genetic engineering approaches have raised the possibility of achieving high levels of resistance to stem borers in rice (Vila et al., 2005). However, such genetic engineering approaches are technically difficult, and their long term effects and safety have not yet been determined (Chow et al., 2016). Screening rice varieties for high levels of resistance to the striped stem borer is an effective strategy for pest management. However, the identification and screening for rice varieties resistant to the striped stem borer have not yet been well studied.

In this study, we screened and identified the resistance traits of 47 rice cultivars to the striped stem borer by artificial infestation in both field and greenhouse experiments. We then adopted the Illumina sequencing platform to construct a transcriptome database for comparative analysis to identify susceptible and resistant rice varieties with or without infestation by striped stem borer larvae. Differentially expressed genes (DEGs) with altered expression between susceptible and resistant varieties were identified and verified by quantitative real-time PCR (RT-qPCR). This is the first data set to select resistant rice varieties and analyze resistance-related genes; this resource provides an important foundation for further understanding of the insect resistance mechanisms of selected resistant varieties and also can help us breed for rice resistance to *C. suppressalis*.

## Materials and Methods

### Insects

Larvae of susceptible *C. suppressalis* to host plant were supplied by the Test Center of Pesticides, Shenyang Chemical Industry Research Institute and were fed on japonica rice seedings in an artificial climate chamber (MLR-352H-PC, Panasonic). The insects had been maintained without insecticide exposure for over 30 generations in the laboratory at 25 ± 1°C, 80 ± 1% RH and a photoperiod of 16:8 (L:D) hours.

### Rice Cultivars

Tested rice cultivars (*n* = 47) including japonica, indica and weedy rice were provided by Rice Research Institute of Shenyang Agriculture University, Shenyang, Liaoning Province of China.

### Resistance Identification

#### Field Identification

The germinated rice seeds were sown on April 21, 2014 and transplanted 1 month later. Forty seven rice cultivars were designated as 47 treatment plots. The rice seedlings were transplanted in triplicate; thus a total of 141 experimental plots were conducted at the Rice Research Institute of Shenyang Agricultural University. Each experimental plot consisted of one row with 10 holes. Holes were spaced 16.7 cm apart and plants within each row were spaced 26.6 cm apart. The highly susceptible cultivar 1665 was used for the control because its tillering ability is stronger than the weedy rice and its growth period is close to the other cultivars (Wang et al., 2015; Wang, 2016). No pesticides were used during the whole growth period of plants.

During the tillering period, an artificial infestation was introduced from July 7 to 8, 2014. The density of infestation was 2 seedlings with one worm, which is according to the standard of the International Rice Research Institute (Pathak et al., 1971) and Zhou’s methods (1985). The primary hatching larvae were inoculated into the ligule between the stem and the second or third leaf sheath. To minimize confounding factors, any natural enemies and other insects on the plants were removed before inoculation and the plants were covered with a 40 mesh cage. At this time, the total number of tillers of each plant was recorded. The number of “dead hearts” was surveyed 30 days later, and the “dead heart” rate was calculated according to methods in a previous study (Wang et al., 2015). The damage index was calculated by the following formula: Damage index = dead heart rate/dead heart rate of control. The resistance levels of rice cultivars are determined according to Pathak et al. (1971) and are shown in Table 1.

**Table 1:** Grading standard for the strength of resistance^∗^.

Resistance grade	Resistance response	Damage index range (dead hearts) %
0	Highly Resistant(HR)	0
1	Resistant(R)	1–20
3	Moderately Resistance (MR)	21–40
5	Endurance (E)	41–60
7	Susceptible (S)	61–80
9	Highly susceptible (HS)	≥81


**^∗^Grading standard of resistance refers to Pathak et al. (1971).**

#### Indoor Identification

Based field identifications, 15 highly representative rice cultivars with similar growth periods were selected for further resistance identification in the greenhouse. The highly susceptible cultivar 1665 was used as the control.

This study was conducted in the Pesticide Creation Team of Shenyang Sinochem Agrochemicals R&D Co., Ltd. No other rice pests were reared and no pesticides were used in the greenhouse during the whole growth period of these plants. The rice was planted using the pot-culture method in the greenhouse at 28 ± 2°C, 70 ∼ 80% RH, and a photoperiod of 16:8 (L:D) hours. On 12th January 2015, the seeds were soaked in water with 6 days for germination, and were then planted in small plastic cups (10 cm in diameter and 12 cm high). Rice seedlings were then transplanted into square pots (18 cm^∗^26 cm^∗^12 cm) with soil after 16 days. Three replicates of each rice cultivar were planted in individual pot (with 8 holes). On March 10, as the rice was in the tillering stage, larvae were artificially introduced as described above. After infestation, each pot was placed 20 cm apart to prevent the larvae from an adjacent pot affecting the results of the experiment by heteroicous. The total number of tillers and “dead hearts” were surveyed, and the “dead heart” rate was calculated as described above.

### Feeding Induction Treatment

Based on resistance identification results both in field and greenhouse, a series of high resistance cultivars (1688), moderate cultivars (1654) and susceptible cultivars (1665) were placed in the greenhouse as described above. The experiment contains 4 replicates. For each replicate, two pots were randomly divided into two groups, one was the control group (with infestation) and the other was the experimental group (with infestation). Two rows (one row was used for artificial infestation and the other one was regarded as a guarding row), each with 4 rice holes were planted in each pot. For the treatment group (CK, for short), a third instar larva of striped stem borer was placed onto the stem of rice when the rice seedlings had grown to the tillering stage. When half of the larva body had drilled into a stem of rice, a label that marked the time and tillering was tagged on the stem. After 24 h of this artificial infestation, the leaf sheath in which the tiller with larva was located was split gently and the larva was removed. The plant was then packaged in silver paper and was subsequently frozen in liquid nitrogen and stored at -80°C for later use. The plant without any artificial infestation was similarly sampled and treated.

### Effect of Diet Adding Rice Plant Powder on the Survival Rate and Larval Weight of *C. suppressalis*

Larval rearing diet was prepared according to Li et al. (2015) with or without adding rice plant powder (145 g) of different cultivars at the tillering stage. Diet was placed in a glass tube (2cm dia., 10 cm long). Newly hatched larvae were inoculated into different diets with a density of 30 larvae/tube. After 10 days, survival rate and larvae weight were determined. Three replicates were run.

### RNA Extraction, cDNA Synthesis and Illumina Sequencing

Frozen samples were ground in liquid nitrogen using a mortar and pestle. Total RNA of 1688, 1654, and 1665 with or without artificial infestation was isolated using the Ultrapure RNA Kit (CWBIO, Beijing, China) according to the manufacture’s protocol and treated with RNase-free DNase I. The quality and concentration of DNase I-treated RNA was determined using a NanoDrop 2000 UV–vis Spectrophotometer (Thermo Scientific, Waltham, MA, United States). mRNA was isolated from the total RNA using magnetic beads with oligo (dT) and sheared into short fragments using fragmentation buffer. The cDNA was then synthesized using the mRNA fragments as templates using Reverse Transcription Kit Prefect (TaKaRa) following the manufacture’s protocol. Short fragments are purified and resolved with EB buffer for end reparation and single nucleotide A (adenine) addition. The short fragments were then connected with sequencing adapters and analyzed by agarose gel electrophoresis. Suitable fragments were enriched by PCR amplification to construct the cDNA library. Six pooled cDNA libraries were constructed using an mRNA-Seq assay for paired-end transcriptome sequencing and sequenced on an Illumina HiSeq2000 system at Beijing Genomics Institute (BGI, Shenzhen, China). The raw data from Illumina deep-sequencing are available in the NCBI Short Read Archive (SRA) (SRP142306).

### Functional Annotations and Deep Analysis of Gene Expression

The raw reads generated by Hiseq2000 were filtered to remove low-quality reads (reads containing adaptor, reads containing >10% unknown nt “N,” and reads with >50% quality value ≤ 10). After filtering, the remaining reads, which were called clean reads, were used for downstream bioinformatics analysis. We used BWA to map clean reads to the entire genome reference^1^ and used Bowtie to gene reference. Clean reads were mapped to the selected references, and the statistics of alignment results were presented for each reference. After clustering, the unigenes were divided into clusters and singletons. Assembled unigenes were subjected to blastx (BLAST, the basic local alignment search tool) alignment (*E*-value < 1e-5) and several protein databases, including blast nt, description, KEGG Orthology, Gene Ontology (GO) analysis (GO Component, GO Function and GO Process) and PCA analysis. GO function of all-unigenes were categorized by Blast2GO, according to molecular function, biological process and cellular component. The genes’ complex biological behaviors were further examined by pathway annotation using KEGG identifiers^2^.

### Differential Gene Expression in Resistant and Susceptible Rice Cultivars With or Without *C. suppressalis* Artificial Infestation

Differential expressed genes (DEGs) were determined based on their expression abundances in different treatment groups. The FPKM method was used to calculate expression levels of genes and to quantify transcript levels among the different samples, which eliminates the influence of different gene lengths. The GO database was used to annotate DEGs and the numbers of DEGs in each GO term were calculated. KEGG pathway analysis of the DEGs was also performed to identify the associated biochemical and signal transduction pathways.

### Real-Time Quantitative PCR (RT-qPCR) Validation

The expression abundance of selected genes was determined by RT-qPCR to verify the transcriptome results. The 18s rRNA was chosen as a housekeeping gene (Kim et al., 2003). Using the Prime3 software tool (Rozen and Helen, 2000), primers were synthesized by Sangon Biotech (Shanghai, China). The sequences of primers used are shown in Table 2.

**Table 2:** Sequences of primers.

Gene	Primer sequence (5′–3′)	Fragment length (bp)	GenBank accession number
18s rRNA	F: 5′-atgataactcgacggatcgc-3′	20	
	R: 5′-cttggatgtggtagccgttt-3′	20	
LOC_Os07g11650	F: 5′-ggcatcagacaagttcgtcctct-3′	23	NP_001059192.1
	R: 5′-gatgtagttccggcacgatgg-3′	21	
LOC_Os03g02050	F: 5′-acctgccactgatccatcca-3′	20	1L6H
	R: 5′-cgaactgaccacccaagcac -3′	20	
LOC_Os01g12020	F: 5′-gtcgtgaagaaattttacgggtca-3′	24	NP_001042421.1
	R: 5′-cttgcaggagaaactttgcagcta-3′	24	
LOC_Os03g59380	F: 5′-ttgtggctgtcgtggtggt-3′	19	NP_001051651.1
	R: 5′-gctccaggcacctgcaca-3′	18	
LOC_Os07g03880	F: 5′-catttaccaacatgaggcccaata-3′	24	NP_001058828.1
	R: 5′-gctagtccctggaggaggatatga-3′	24	
LOC_Os06g10790	F: 5′-gaacatgtacgttgggttctcgtc-3′	24	NP_001057114.1
	R: 5′-cgaacttgatgatgagcagcttg-3′	23	
LOC_Os07g18230	F: 5′-gcacccaaatctgtaagaccactg-3′	24	NP_001175141.1
	R: 5′-gctggagcaggactattcatagca-3′	24	
LOC_Os10g39700	F: 5′-aggggtagttctgaccagttccag-3′	24	NP_001065197.1
	R: 5′-agttcgatcgcgtgactttcataa-5′	24	
LOC_Os08g41100	F: 5′-ccatgcaaatcgcatacaactaca-3′	24	NP_001055478.1
	R: 5′-atgttcgtggtgagcccgta-3′	20	
LOC_Os07g18230	F:5′-gcacccaaatctgtaagaccactg-3′	24	NP_001175141.1
	R: 5′-gctggagcaggactattcatagca-3′	24	
LOC_Os07g04220	F: 5′-tgacatcaccaccagtagcagtgt-3′	24	BAC83513.1
	R: 5′-aacctgggatccactctcaaagac-3′	24	
LOC_Os01g12160	F: 5′-tcttccctttgccgctcttc-3′	20	EEE54131.1
	R: 5′-tcctcgtcgccttcttctcc-3′	20	
LOC_Os03g15880	F: 5′-agtcttaacgatttccggcttgtc-3′	24	NP_001049649.1
	R: 5′-agactccccaacattcccaagtaa-3′	24	
LOC_Os05g37690	F: 5′-accactgaagatcactgccaactc-3′	24	NP_001055700.1
	R: 5′-ggcagctatgtattccagttcacg-3′	24	
LOC_Os09g26780	F: 5′-ggctcaacagctgaccatcttcta-3′	24	NP_001063273.1
	R: 5′-cattcatcctgccctttctcttct-3′	24	
LOC_Os02g34320	F: 5′-accaggccttcatcaacgtgt-3′	21	EEC73367.1
	R: 5′-tatctccgagacagccagttggta-3′	24	
LOC_Os04g51070	F: 5′-tactttgaggattcccacgatcaa-3′	24	NP_001053749.1
	R: 5′-tgctcttgcagcttcagctagatt-3′	24	
LOC_Os03g56950	F: 5′-ctccagctatgaacccaatgaatg-3′	24	EEC76276.1
	R: 5′-cggtatctggttttgctgtgctac-3′	24	
LOC_Os11g32100	F: 5′-atcaaggaagagatctgcccaagt-3′	24	NP_001067987.2
	R: 5′-gccattgaagcaactgattacagc-3′	24	
LOC_Os12g02420	F: 5′-gcaagtccagcagaaggacactaa-3′	24	NP_001065590.1
	R: 5′-gtgaattgaagcagatcgaaatgg-3′	24	
LOC_Os01g09080	F: 5′-tagggtttccgtcagagtcaagtg-3′	24	NP_001042242.1
	R: 5′-tacgtcgtgatcaggatcgacat-3′	23	
LOC_Os08g09900	F: 5′-attccaccctttctcaccattcat-3′	24	NP_001061202.1
	R: 5′-gaattgtactctgcccacacatcc-3′	24	
LOC_Os03g58420	F: 5′-gatcagcttcagcttcgacaactc-3′	24	EEC76340.1
	R: 5′-ccttcttctccctgggttcttct-3′	24	
LOC_Os08g03690	F: 5′-aatccaatcttcttggtgccatt-3′	24	EAZ05489.1
	R: 5′-gccgtcgtcttcttcttcttcttc-3′	24	


The expression patterns of candidate genes were analyzed by RT-qPCR using a Bio-Rad CFX96 (Bio-Rad, Hercules, CA, United States). Each reaction contained 12.5 μl of 2 × SYBR Premix Ex Taq II (TaKaRa), 2 μl of cDNA, and 1 μM of gene-specific primers in a final volume of 25 μl. The RT-qPCR was biologically repeated three times with independently synthesized cDNA. Three technical replicates were used for each sample. A negative control using RNase-free water instead of cDNA and a no transcription control were also conducted. Relative expression levels of each gene were calculated using the 2^-ΔΔCt^ algorithm. In addition, a correlation analysis between RNA-Seq and RT-qPCR results was conducted.

### Data Analysis

Results are reported as mean ± standard deviation (SD). The data for resistance identification was analyzed by one way analysis of variance (ANOVA, *P* < 0.05). Fisher’s least significant difference (LSD) test was used to separate means (*P* < 0.01). The software SPSS v17.0 (IBM Inc., Chicago) was used for data analyses.

## Results

### Resistance Identification

#### Field Identification

After 30 days of artificial infestation, all rice cultivars demonstrated the symptoms of “dead heart” except the rice cultivar 1688. The dead heart rate of 47 rice cultivars ranged from 0 (1688) to 43.5% (1677) and the damage index ranged from 0 to 87.33% (Table 3). The variance analysis results showed that there was a significant difference between different cultivars (*P* < 0.001; *F* = 3.085; *df* = 46, 94). One highly resistant (HR) cultivar (1688), 3 resistant cultivars (Z46, 1611 and 1689), 13 moderately resistant (MR) cultivars, 15 endurance cultivars, 12 susceptible cultivars and 2 highly susceptible cultivars (Z52 and 1677) were determined according to their resistance grades (Table 4). The control cultivar 1665 exhibits the highest dead heart rate (49.81%). The 13 MR cultivars are 1610, Z48, 1654, 1663, 1683, 1681, 1676, Z43, 1660, Z47, 1682, 1687, and 1678. The high endurance cultivars are 1669, 1656, 1661, 1690, 1670, 1609, 1572, 1685, 1565, 1571, 1659, 1655, 1686, 1679, and Z50. The 12 susceptible cultivars are 1668, 1657, 1674, 1684, 1664, 1667, Z49, 1666, 1612, 1652, 1653, and Z51 (Table 3).

**Table 3:** Identification results of rice resistance to SSB in field.

Cultivars	Rate of dead hearts%	Damage index%	Resistance grade	Resistance response	Types
1688	0	0	0	HR	Japonica rice
Z46	5.42	10.87	1	R	Weedy rice
1611	7.79	15.64	1	R	Indica type rice
1689	9.51	19.09	1	R	Japonica rice
1610	13.69	27.48	3	MR	Indica type rice
Z48	13.70	27.51	3	MR	Weedy rice
1654	15.28	30.67	3	MR	Japonica rice
1663	16.59	33.31	3	MR	Japonica rice
1683	16.67	33.46	3	MR	Japonica rice
1681	17.51	35.16	3	MR	Japonica rice
1676	17.55	35.24	3	MR	Japonica rice
Z43	18.33	36.80	3	MR	Weedy rice
1660	18.33	36.80	3	MR	Japonica rice
Z47	19.17	38.48	3	MR	Weedy rice
1682	19.44	39.03	3	MR	Japonica rice
1687	19.78	39.71	3	MR	Japonica rice
1678	19.91	40.00	3	MR	Japonica rice
1669	20.87	41.89	5	E	Japonica rice
1656	20.89	41.93	5	E	Japonica rice
1661	23.81	47.80	5	E	Japonica rice
1690	24.17	48.51	5	E	Japonica rice
1670	24.52	49.23	5	E	Japonica rice
1609	24.68	49.53	5	E	Indica type rice
1572	25.00	50.19	5	E	Indica type rice
1685	25.26	50.72	5	E	Japonica rice
1565	25.56	51.30	5	E	Indica type rice
1571	26.38	52.95	5	E	Indica type rice
1659	26.70	53.59	5	E	Japonica rice
1655	27.78	55.76	5	E	Japonica rice
1686	28.99	58.19	5	E	Japonica rice
1679	29.65	59.51	5	E	Japonica rice
Z50	30.00	60.22	5	E	Weedy rice
1668	30.22	60.66	7	S	Japonica rice
1657	30.56	61.34	7	S	Japonica rice
1674	32.51	65.25	7	S	Japonica rice
1684	32.87	66.00	7	S	Japonica rice
1664	35.00	70.26	7	S	Japonica rice
1667	35.32	70.90	7	S	Japonica rice
Z49	35.45	71.17	7	S	Weedy rice
1666	35.56	71.38	7	S	Japonica rice
1612	37.58	75.44	7	S	Indica type rice
1652	38.91	78.10	7	S	Japonica rice
1653	39.68	79.66	7	S	Japonica rice
Z51	39.68	79.66	7	S	Weedy rice
Z52	41.67	83.64	9	HS	Weedy rice
1677	43.50	87.33	9	HS	Japonica rice
1665(CK)	49.81	100.00	9	HS	Japonica rice


*HR, highly resistant; R, resistant; MR, moderately resistant; E, endurance; S, susceptible; HS, highly susceptible.*

**Table 4:** Identification result of rice resistance to SSB in greenhouse.

Cultivars	Rate of dead hearts%	Damage index%	Resistance grade	Resistance response	Types
1688	5.56	6.47	1	R	Japonica rice
1654	33.41	38.92	3	MR	Japonica rice
Z46	41.27	48.08	5	E	Weedy rice
1689	43.13	50.24	5	E	Japonica rice
1611	42.50	52.43	5	E	Indica type rice
Z43	50.00	58.25	5	E	Weedy rice
1663	57.14	66.57	5	E	Japonica rice
Z48	56.88	66.26	7	S	Weedy rice
1669	58.33	67.96	7	S	Japonica rice
1653	63.75	74.27	7	S	Japonica rice
1656	63.75	74.27	7	S	Japonica rice
1660	68.33	79.61	7	S	Japonica rice
Z52	81.89	95.41	9	HS	Weedy rice
1677	96.67	112.63	9	HS	Japonica rice
1665(CK)	85.83	100.00	9	HS	Japonica rice


**R, resistant; MR, moderately resistant; E, endurance; S, susceptible; HS, highly susceptible*.*

#### Indoor Identification

Based on the results of field identification, 15 highly representative rice cultivars (Table 4), including 1688, were selected for further resistance identification in the greenhouse. Results further showed that 1688 is a resistant cultivar, with the lowest dead heart rate of 5.56%, whereas the control cultivar 1665 exhibits a 85.83% dead heart rate (Table 4). The highly susceptible cultivar1677 determined by field identification also performed as a susceptible cultivar, and had the highest rate of dead hearts (112.63%) in 15 rice cultivars (Table 4). Combining the results of field identification with indoor identification, 1688 is a resistant cultivar and 1654 is a MR cultivar.

### Effect of Diet Adding Rice Plant Powder on the Survival Rate and Larval Weight of *C. suppressalis*

Statistics suggest that the *C. suppressalis* larvar survival rate of rearing with basal diet + 1665 powder was significant higher (*P* < 0.05) than that rearing with basal diet, basal diet + 1688 powder, and basal diet + 1654 powder. The larvae weight of *C. suppressalis* was significant higher (*P* < 0.05) when rearing with basal diet than those rearing with basal diet adding rice plant powder; larvae weight of *C. suppressalis* rearing with basal diet + 1688 powder was significant higher (*P* < 0.05) than those rearing with basal diet adding 1665 or 1654 rice plant powder (Supplementary Table S1).

### Results of RNA-Seq

After the total RNA extraction and DNase I treatment, magnetic beads with Oligo (dT) were used to isolate mRNA (for eukaryotes) or by removing rRNAs from the total RNA (for prokaryotes). Mixed with the fragmentation buffer, the mRNA was fragmented into short fragments. Then cDNA was synthesized using the mRNA fragments as templates. Short fragments were purified and resolved with EB buffer for end reparation and single nucleotide A (adenine) addition. After that, the short fragments were connected with adapters. After agarose gel electrophoresis, the suitable fragments were selected for the PCR amplification as templates. During the QC steps, the Agilent 2100 Bioanaylzer and ABI StepOnePlus Real-Time PCR System were used in quantification and qualification of the sample library. Six cDNA libraries were prepared from three cultivars of 1688, 1654, and 1665, which included the control group (CK) and Artificial Infestation treatment (AI) for each cultivar. Then the six cDNA libraries were subjected to Illumina sequencing. Illumina paired-end sequencing generated a total of 295.5 million raw reads. After cleaning and quality checks, 293.6 million clean reads (14.8 Gb) were obtained, with an average of 48.9 million reads (∼2.5 Gb) per sample. The 1654AI, 1654CK, 1665AI, 1665CK, 1688AI, and 1688CK cDNA libraries generated 49,054,738, 48,913,078, 49,011,744, 48,730,342, 48,937,446, and 48,997,360 clean reads, respectively (Table 5). The sample of 1654AI has the highest clean reads (49,054,738) and genome map rate (86.98%). The highest rate of genome map is 84.61% (1688AI) and the lowest rate is 83.09% (1654AI).

**Table 5:** Sequence and assembly summary of transcriptome.

Sample name	Clean reads	Genome map rate	Gene map rate	Expressed gene	Expressed transcripts
1654AI	49054738	86.98%	83.09%	30060	37608
1654CK	48913078	85.79%	83.34%	29289	36616
1665AI	49011744	85.94%	84.16%	27961	34915
1665CK	48730342	85.22%	83.78%	29569	36814
1688AI	48937446	84.54%	84.61%	29803	36880
1688CK	48997360	85.63%	84.40%	29897	37134


*AI, artificial infestation; CK, control treatment group without artificial infestation.*

### Number and Expression Levels of Genes Among Three Cultivars

**FIGURE 1:**
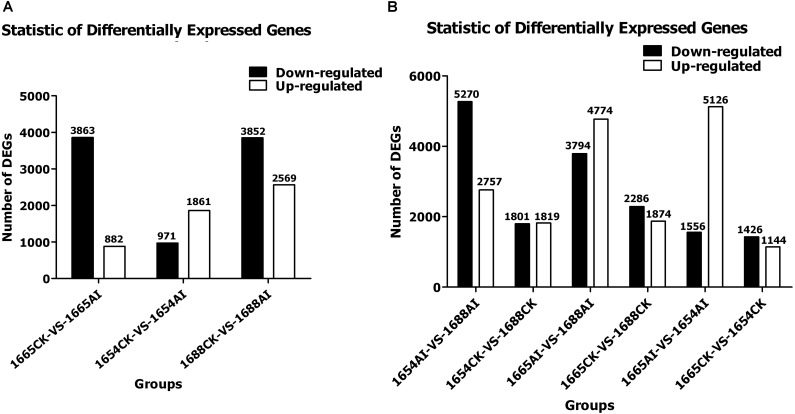
Statistics of differentially-expressed genes in three rice cultivars after SSB artificial infestation compared with the non-infested control: **(A)** within; and **(B)** between cultivars. (AI) artificial infestation; (CK) non-infested control.

The 1654AI, 1654CK, 1665AI, 1665CK, 1688AI, and 1688CK cDNA libraries generated 30,060, 29,289, 27,961, 29,569, 29,803, and 29,897 expressed genes, respectively (Table 5) and 37,608, 36,616, 34,915, 36,814, 36,880, and 37,134 expressed transcripts, respectively (Table 5). Genes with an adjusted *p*-value of <0.05 found by DESeq were assigned as differentially expressed. There were 1,144, 1,874, and 1,819 up-regulated genes and 1,426, 2,286, and 1,801 down-regulated genes in 1665-VS-1654, 1665-VS-1688, and 1654-VS-1688, respectively. After artificial infestation with *C. suppressalis*, the resistant (R) cultivar 1688 had 4,774 up-regulated genes and 3,794 down-regulated genes compared with the susceptible (S) cultivar 1665. The MR cultivar 1654 had 5,126 up-regulated genes and 1,556 down-regulated genes when compared with 1665. Moreover, in the group of 1654AI-VS-1688AI, there were 2,757 up-regulated genes and 5,270 down-regulated genesin 1654 compared with the 1665 (Figure 1).

### Number and Expression Levels of Genes Between Different Treatments

Using DESeq software to analyze the expression level of differential genes, a total of 6,421 DEGs with 2,569 up-regulated genes and 3,852 down-regulated genes were identified in 1688 after artificial infestation with *C. suppressalis*, compared with the non-infested control. For 1654, only 1,861 genes were up-regulated and 971 genes were down-regulated after artificial infestation with *C. suppressalis* compared to the non-infested control. For susceptile cultivar 1665, a total of 4,745 DEGs were identified, but only 882 genes were up-regulated and 3,863 genes were down-regulated after artificial infestation with *C. suppressalis* compared to the non-infested control (Figure 1A). As shown in Figure 1, the differentially-expressed genes in the group of 1688CK-VS-1688AI are more than in the groups 1665CK-VS-1665AI and 1654CK-VS-1654AI. When comparing 1654AI and 1688AI, there are 5,270 down-regulated genes. In other words, cultivar 1688 has 5,270 down-regulated genes with cultivar 1654 after artificial infestation which had 1,801 down-regulated genes before that (Figure 1). Those changes among all the groups may be caused by feeding induction. The stress response of moderate cultivar 1654 to feeding induction is less significant than for the other two cultivars (1688 and 1665) (Figure 1).

### Possible Genes Related to *C. suppressalis* Resistance

**FIGURE 2:**
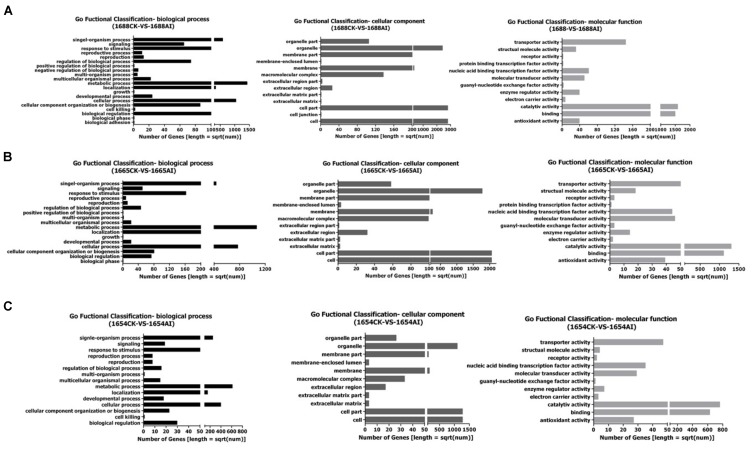
Gene Ontology (GO) Functional Classification: **(A)** 1688CK-vs-1688AI; **(B)** 1665CK-vs-1665AI; **(C)** 1654CK-vs-1654AI.

A deep analysis based on DEGs, including Gene Ontology (GO) enrichment analysis, pathway enrichment analysis and so on, was conducted. All the differentially-expressed genes of sequencing samples in GO enrichment analysis are divided into three main categories: biological processes, cellular components and molecular functions (Figure 2).

The DEGs are divided into 45 sub-categories, including 20, 13 and 12 sub-categories in biological processes, cellular components, and molecular functions, respectively in the cultivar 1688 after artificial infestation (Figure 2A). The DEGs in 1665 after artificial infestation are divided into 41 sub-categories belonging to three main GO categories, including 17 in biological processes, 12 in cellular components and 12 in molecular function sub-categories, respectively (Figure 2B). The DEGs in 1654 after artificial infestation are divided into 37 sub-categories belonging to three main GO categories, including 15 in biological processes, 11 in cellluar components and 11 in molecular function sub-categories, respectively (Figure 2C). Figure 2 showed that those DEGs were mainly enriched in the biologicalprocess group. Those DEGs were mainly involved in functions of the metabolic process, biological regulation, cellular processes, localization and signaling. These results indicate that the majority of DEGs in response to *C. suppressalis* feeding might be related to various metabolic processes, implying the resistance of rice involves changes of metabolites.

**FIGURE 3:**
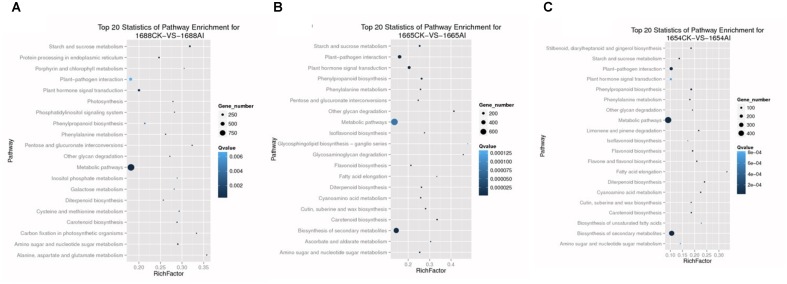
Top 20 statistics of pathway enrichment for each group: **(A)** 1688CK-vs-1688AI; **(B)** 1665CK-vs-1665AI; **(C)** 1654CK-vs-1654AI. (Vertical axis represents the name of the pathway, while the horizontal axis represents the rich factor. The greater the rich factor, the greater the degree of enrichment. The size of the point indicates the number of DEGs in this pathway, and the color of the points corresponds to different *Q*-value ranges. The values of *Q* were between 0 and 1. The closer the *Q*-value is to zero, the more significant the enrichment).

We also used KEGG to analyze the pathways of the DEGs. KEGG provides the integration of pathways, such as metabolic pathways, plant–pathogen interaction pathways and plant hormone signal transduction pathways, in which the DEGs are involved. In the pathway enrichment analysis, 66,360 differentially-expressed genes of samples were annotated. A total of 22,120 differentially-expressed genes of cultivar 1688 were annotated using pathway enrichment analysis, which consisted of 125 pathways. There were 121 pathways in cultivar 1654 which were the same as in cultivar 1665. Using a probability of false discovery rate (FDR) of ≤0.05, the top 20 statistics for pathways are shown in Figure 3. These were significantly enriched in each cultivar in response to *C. suppressalis* feeding. In addition, the plant hormone signal transduction pathway and plant–pathogen interaction pathway, which involve jasmonic acid (JA), salicylic acid (SA), ethylene (ET), mitogen-activated protein kinase (MAPK) and the WRKY family, all play an important role in rice resistance (Eulgem et al., 2000; Zheng et al., 2006; Pandey and Somssich, 2009; Wu and Baldwin, 2010; Arimura et al., 2011; Birkenbihl et al., 2012; Erb et al., 2012; Fu et al., 2012; Chujo et al., 2013; Wu et al., 2012; Wei et al., 2013). Therefore, it is interesting to investigate DEGs within these two pathways.

### Validation the Expression of DEGs Using RT-qPCR

A total of 24 DEGs (*P*-values < 0.01) were screened as candidate genes to test theirpossible role in *C. suppressalis* resistance. DEGs that were significantly enriched for resistance and *C. suppressalis* feeding induction were investigated using RT-qPCR. These 24 candidate genes include 5 proteinase inhibitor genes (LOC_Os07g11650, LOC_Os03g02050, LOC_Os01g12020, LOC_Os03g59380, and LOC_Os08g03690), 3 lectin genes (LOC_Os07g03880, LOC_Os06g10790, and LOC_Os07g18230), 3 chitinase genes (LOC_Os10g39700, LOC_Os08g41100, and LOC_Os05g33150), 9 genes in the plant hormone signal transduction pathway (LOC_Os07g04220, LOC_Os01g12160, LOC_Os03g15880, LOC_Os05g37690, LOC_Os09g26780, LOC_Os02g34320, LOC_Os04g51070, LOC_Os03g56950, and LOC_Os11g32100) and 4 genes in plant-pathogen interaction pathway (LOC_Os12g02420, LOC_Os01g09080, LOC_Os08g09900, and LOC_Os03g58420).

**FIGURE 4:**
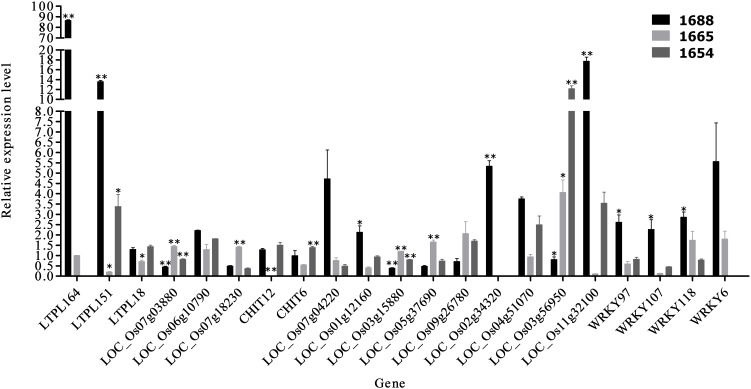
The test results of RT-qPCR of 21 candidate genes. [21 candidate genes were analyzed by RT-qPCR. Relative expression levels of each gene were calculated using the 2–ΔΔCt algorithm. The software SPSS v17.0 (IBM Inc., Chicago) and the GraphPad prism 5 were used for data analyses and this figure. Then data are presented as mean fold change ± standard error (SE) and indicated by vertical bars (^∗^*P* < 0.05, ^∗∗^*P* < 0.01). The 18s rRNA was chosen as a housekeeping gene (F: 5′-atgataactcgacggatcgc-3′; R: 5′-cttggatgtggtagccgttt-3′)].

Of the 24 candidates identified from RNA-Seq differential expression analysis, 21 were verified using RT-qPCR. Statistical analysis of the RT-qPCR data revealed that 13 genes were significantly up-regulated and 8 were significantly down-regulated in the artificially infested group of resistant cultivar 1688 (1688AI) compared to the non-infested control group of 1688 (1688CK) (Figure 4). These results are in accord with those of RNA-Seq. Three genes showed a more than 10-fold higher expression in 1688AI than in 1688CK: *LTPL164* (86-fold), *LTPL151* (13-fold), and *LOC Os11g32100* (17-fold) (Figure 4).

**FIGURE 5:**
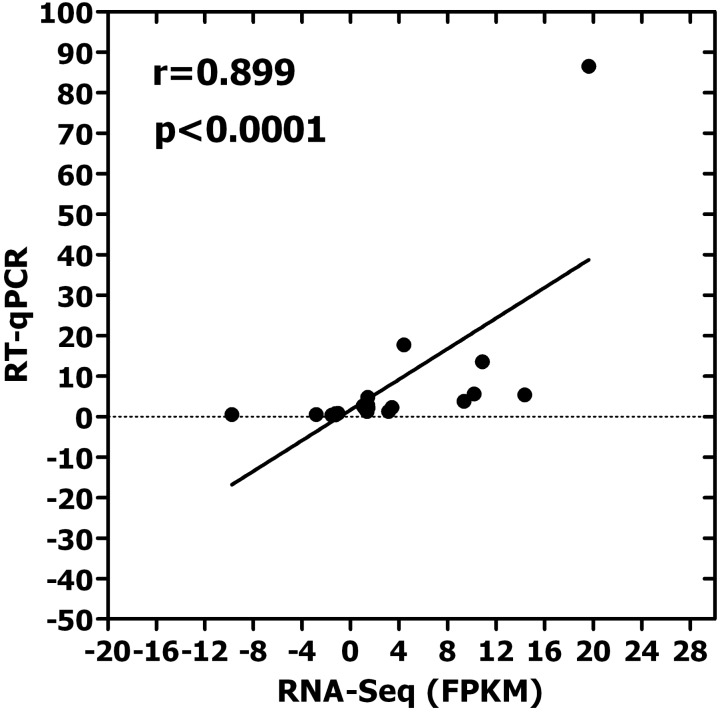
Correlation analysis for 21 candidate genes between RNA-Seq and RT-qPCR (*r* = 0.899, *p* < 0.001). [Both of software SPSS v17.0 (IBM Inc., Chicago) and the GraphPad prism 5 were all used for data analyses and the figure was made by Graphpad prism 5. The *X*-axis represents the value of FPKM in RNA-Seq, which was used in calculated expression level. The *Y*-axis shows the relative expression level of 21 candidate genes in RT-qPCR. The result of correlation analysis shows that there is a high correlation between RNA-Seq and RT-qPCR results. It is suggests that the results of RNA-Seq and RT-qRCR were consistent, and also suggests that the selected DEGs maybe involved in insect resistance. These 21 candidate genes are LTPL 164, LTPL151, LTPL18, LOC_Os07g03880, LOC_Os06g10790, LOC_Os07g18230, CHIT12, CHIT6, LOC_Os07g04220, LOC_Os01g12160, LOC_Os03g15880, LOC_Os05g37690, LOC_Os09g26780, LOC_Os02g34320, LOC_Os04g51070, LOC_Os03g56950, LOC_Os11g32100, WRKY97, WRKY107, WRKY118, and WRKY6, respectively].

A correlation analysis between RNA-Seq and RT-qPCR results was conducted. The high correlation (*r* = 0.899, *P* < 0.0001) between RNA-Seq and RT-qPCR results (Figure 5) suggests that the results of RNA-Seq and RT-qRCR were consistent, and also suggests that the selected DEGs maybe involved in insect resistance.

## Discussion

### Resistance Identification

In this study, the resistance traits of 47 rice cultivars from three different types (japonica rice, indica type rice, and weedy rice) to the striped stem borer were determined by field identification and indoor identification. Using a resistance identification method proposed by Zhou (1985), Shu et al. (2003), cultivar 1688 was identified as a HR cultivar in the field identification trial, but indoor trial result suggests that it a resistant cultivar.

**FIGURE 6:**
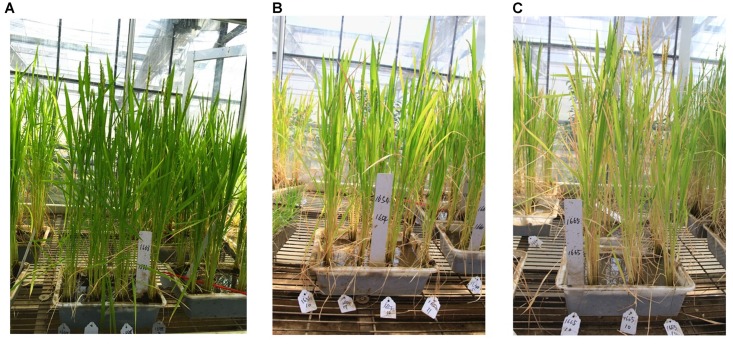
Comparison of the growth among 1688, 1654, and 1665. **(A)** Growth status of 1688 after 30 days of artificial infestation. **(B)** Growth status of 1654 after 30 days of artificial infestation. **(C)** Growth status of 1665 after 30 days by artificial infestation. (Method: a third instar larva of striped stem borer was placed onto the stem of rice when the rice seedlings had grown to the tillering stage. When half of the larva body had drilled into a stem of rice, a label that marked the time and tillering was tagged on the stem. After 24 h of this artificial infestation, the leaf sheath in which the tiller with larva was located was split gently and the larva was removed. The plant was then packaged in silver paper and was subsequently frozen in liquid nitrogen and stored at -80°C for later use. The plant without any artificial infestation was similarly sampled and treated).

In this study, the dead heart rates for control cultivar 1665 are different between field identification (49.81%) and indoor identification (85.83) trails (Figure 6). The different results of resistance identification between field identification and indoor identification are similar with the result of Zhou’s research (1985). The possible reason for such a large difference may be the fact that the method can be easily affected by many factors, such as the density of plants, spacing of experiments, climate conditions and so on (Luo et al., 2006). Compared with field conditions, the greenhouse has suitable temperatures and a mild climate, with no natural enemies and other insects. As a result, the survival rate and the boring rate of larvae were increased in the greenhouse, and the larvae transferred frequently to find suitable boring spaces after artificial infestation. In addition, the pot-culture method limited the thickness of the soil and therefore the absorption rates of water, nitrogen and phosphorus decreased, causings lower rice growth. Therefore, the dead heart rate and damage index are higher in the greenhouse than in the field.

In this study, we found that japonica rice is more resistance to striped rice borer than is indica rice. Eleven of 32 (34.4%) japonica rice units were found to be resistant (R), MR or HR to striped rice borer. But this rate was 28.6% (2 of 7) in indica rice. This result is in line with previous research (Zhou, 1985; Gu et al., 1989; Hao, 2011). Previous studies have found that the damage of SSB is closely related to the width of a leaf, the degree of compactness of the sheath, and the vascular bundle interval, etc. (Hao, 2011). Thin short stems and appressed sheaths are all adverse factors for boring or the growth of larvae, which in turn can cause growth and developmental delays and a high mortality rate (Gu et al., 1989). Compared with japonica rice, indica type rice is more sensitive because it has thick and strong stems and loose sheaths, etc. (Zhou, 1988). In addition, the HR cultivar 1688 and MR cultivar 1654 observed in this study have erect and lodging-resistant stems, more grains and a higher density of leaf-trichomes. This might be adverse to attack by striped rice borer. Actually, we found that some of the larvae died in the process of drilling. Our results suggest that 1688 and 1654 can be cultivated as striped rice borer resistant cultivars in the rice-planting regions of Northeast China.

### RNA-Seq

Although the experimental design was reasonable and its operation was normative, there are still some drawbacks in this work for reducing the error of research results. Firstly, no time gradients were set for feeding induction. Rice samples were artificially infested with striped rice borer for 24 h and then rice plants were sampled and sequenced. In other words, the results of RNA-Seq reflect the changes of gene expression during the 24-h period. If there were time gradients such as 0, 3, 6, 12, 24, and 48 h, results could be more comprehensive and differentially-expressed genes and pathways could be analyzed more accurately. Secondly, a mechanical damage study could be conducted. Daily cultivation management, investigation techniques, artificial infestation or sampling may cause mechanical damage, resulted in changes in gene function and metabolic pathways. Therefore, artificial mechanical damage should be carried out in the future research. Although no biological replicate was conducted for RNA-Seq, each treatment was sampled in a mixed pool and was RNA-sequenced. A correlation analysis between RNA-Seq and RT-qPCR was conducted; the high correlation suggests that the results of RNA-Seq and RT-qPCR were consistent.

### Screening of DEGs

#### Proteinase Inhibitor Genes

Proteinase inhibition plays an important role in plant defense systems that can inhibit the activity of substances such as trypsin and chymotrypsin, etc. In 1989, Johnson found that the transgenetic tobacco plants carrying the *pi-I* gene and *pi-II* gene all showed a good ability to resist insects (Johnson et al., 1989). In this category, the LTPL164, LTPL151, LTPL28, LTPL24, and LTPL18 gene expression levels increased. These five candidate genes belong to the cytoplasmic vesicle of the cellular component in the GO enrichment analysis. Meanwhile, LTPL151, LTPL28, and LTPL24 also belong to the binding of molecular function and localization of biological processes. LTPL 18 is involved in both of starch and sucrose metabolism pathways. LTPL164, LTPL24, and LTPL18 are the significant differentially-expressed genes of the HR cultivar 1688 (*P* < 0.01), but they are not significant in the moderate cultivar 1654 and highly susceptible cultivar 1665 (*P* > 0.05). The gene expression of LTPL164 and LTPL24 was 0 FPKM in 1688CK; then the expression level increased after artificial infestation (6082 FPKM and 5 FPKM, respectively). The expression of LTPL151 were 1FPKM in 1688CK and 1850 FPKM in 1688AI which was not a significant differentially-expressed gene of 1665 (*P* > 0.05); the expression level of this gene was up-regulation from 1654CK to 1654AI. The expression level of the candidate gene of LTPL28 increased in cultivar 1688 and cultivar 1654 after artificial infestation, but decreased in cultivar 1665. These changes of data may be caused by induced feeding.

#### Lectin Genes

*Galanthus Nivalis* Agglutinin (GNA) is one of the lectin genes, which is most recognized now (Zhang et al., 2003, 2005, 2010; Feng et al., 2006; Wang and Fang, 2014). Research has shown that GNA can inhibit the growth of aphids and provides moderate resistance to Lepidoptera. GNA also has a significantly toxic effect on rice leafhopper (*Nephotettix cinciteps*), whitebacked planthopper (*Sogatella furcifera*), rice brown planthopper (*Nilaparvata lugens*) and so on (Xue et al., 2008). In this study, there are 3 significant differentially-expressed genes selected as candidate genes in the group of 1688CK-VS-1688AI (LOC_Os07g03880, LOC_Os06g10790, and LOC_Os07g18230, respectively). They are not only classified as cytoplasmic vesicle of cellular component and protein kinase of molecular function, but also in the both plant hormone signal transduction pathway and the plant-pathogen interaction pathway. LOC_Os06g10790 is the up-regulated significant differentially-expressed gene of cultivar 1688, but is not significant in cultivars 1654 and 1665. LOC_Os07g03880 and LOC_Os07g18230 are down-regulated genes of 1688 and 1654, which are up-regulated genes of 1665.

#### Chitinase Genes

Wheat α-amylase inhibitors and pea α-amylase are the focus of current research. The mortality rate increases by 30–40% after feeding lepidopterous larvae with the tobacco leaves of transgenic wheat α-amylase inhibitor gene (Chen et al., 2008). Shade found that transgenic pea seeds expressing the alpha-amylase inhibitor of the common bean were resistant to bruchid beetles, and could significantly inhibit the growth of those insects (Shade et al., 1994). LOC_Os10g39700, LOC_Os08g41100, and LOC_Os05g33150 were selected in this part. In the group of 1688CK-VS-1688AI, LOC_Os10g39700, and LOC_Os08g41100 are the significant differentially-expressed genes but are not the significant ones of cultivars 1665 and 1654. LOC_Os05g33150 is the down-regulated differentially-expressed gene in both cultivars 1688 and 1665, but is the reverse in the MR cultivar 1654.

#### Plant Hormone Signal Transduction Pathway

Induced feeding can stimulate a plant to synthesize signaling molecules and pathways, such as JA, SA, ET, and MAPK etc. The transmission of signaling molecules can induce efficient transcription expression of defense-related genes and produce and release a large amount of volatile organic compounds, which can help parasitic or predatory natural enemies to locate insects and effectively control feeding and spawning of insects (Wu and Baldwin, 2010; Arimura et al., 2011; Erb et al., 2012). JA plays a significant role in inducing synthesis and signaling transmission. For this reason, a plant hormone signal transduction pathway that begins with α-linolenic acid, produces JA and eventually produces a stress response may participate in insect resistance. In this pathway, 9 candidate genes were selected in which the expression level in cultivar 1688 (up-regulated) was contrary to that in cultivars 1665 or 1654.5 of 9 candidate genes were up-regulated and another 4 candidate genes were down-regulated.

#### Plant–Pathogen Interaction Pathway

WRKY25 and WRKY29 of the WRKY family participate in and produce defense-related gene induction in two pathways of the plant–pathogen interaction pathway. In recent years, a few studies have reported that the WRKY family plays an important role in mechanisms of plant insect-resistant, in which the WRKY family has been studied and contributes much more in plant disease resistance (Van Eck et al., 2010; Atamian et al., 2012). As a result, four significant differentially-expressed genes (WRKY97, WRKY107, WRKY118, and WRKY6) in cultivar 1688 were selected as candidate genes in this pathway, all of which were up-related genes. These candidate genes were not the significant differentially-expressed genes of cultivar 1654. And they were not significant differentially-expressed genes except LOC_Os12g02420 (down-regulated) in cultivar 1665.

In the process of screening candidate genes, the plant-pathogen interaction pathway was selected. In this pathway, there are two biological pathways that the WRKY family is involved in and result in defense-related gene induction. Previously, studies found that the WRKY family transcription factor can regulate plants’ biological and abiological stress to acquire resistance (Rushton et al., 2015). Currently, most of the WRKY family transcript factors that have been studied are associated with disease-resistant properties. For example, the WRKY33 transcription factor performs positive regulation to acquire resistance to *Alternaria brassicicola* in *Arabidopsis*; the WRKY family transcription factors can increase the resistance to *A. brassicicola* (Eulgem et al., 2000; Zheng et al., 2006; Pandey and Somssich, 2009; Birkenbihl et al., 2012; Fu et al., 2012; Wu et al., 2012; Chujo et al., 2013; Wei et al., 2013). In recent years, a few studies reported changes of expression levels in rice after feeding by herbivorous insects (Zhou et al., 2011). WRKY3 and WRKY6 have been found to produce significant resistance to tobacco hawkmoth (*Manduca sexta*) in tobacco (*Nicotiana attenuata*) (Skibbe et al., 2008). Thus, the significant differentially-expressed genes of WRKY97, WRKY107, WRKY118, and WRKY 6 in the HR cultivar 1688 were selected as candidate genes for further research.

### RT-qPCR Verification

In this part of study, the expression trend of 21 candidate genes were similar to the results of RNA-Seq on the HR cultivar 1688, the MR 1654 and highly susceptible cultivar 1665, indicating that the expression of the 21 candidate genes are reliable. However, we did not acquire the RT-qPCR data for three genes LTPL 24, LTPL 28 (proteinase inhibitor genes) and CHIT15 (chitinase gene). The possible reason for this RT-qPCR failure may be the low expression level of these genes. RNA-Seq results indicate that the expression of LTPL24 is only 5 FPKM in 1688AI and 0 FPKM in 1688CK.

In this study, three genes *OsLTPL164*, *OsLTPL151*, and *LOC Os11g32100* showed a more than 10-fold higher expression in 1688 under *C. suppressalis* infestation compared to that without *C. suppressalis* infestation. Our previous studies have demonstrated that both *OsLTPL164* and *OsLTPL151* genes are involved in resistance to *C. suppressalis* in rice based on the tissue-specific expression patterns and expression profiles in *C. suppressalis* infestation and mechanical damage treatment in three conventionally grown rice lines 1654, 1665, and 1688 (He et al., 2018). The expression level of *OsLTPL164* and *OsLTPL151* in stem and leaf was significantly higher than that in root after *C. suppressalis* infestation (He et al., 2018). This result was probably associated with the feeding behavior of *C. suppressalis* in host plant. Based on our observation, the newly-hatched larvae firstly wandered and fed for a period of time (about 30 min) on rice leaves, then they found a suitable borer site and bored into the stem of rice for feeding. Therefore, the higher expression level of *OsLTPL164* and *OsLTPL151* genes in leaf and stem may suggest a potential role of these genes in resistance to *C. suppressalis*.

## Conclusion

In summary, we identified the resistance traits of 47 rice cultivars to the striped stem borer by artificial infestation in both field and greenhouse experiments. We then used RNA-Seq to obtain comprehensive sequences from identified susceptible and resistant rice cultivars, and those cultivars with or without striped stem borer larvae infestation. From the comparison of transcriptomes, we identified several possible pathways associated with striped stem borer resistance and feeding induction and further verified the transcript level of genes belonging to those pathways. Our results suggest that induced expression of proteinase inhibitor, lectin and chitinase gene families, and plant hormone signal transduction and plant-pathogen interaction pathways are involved in striped stem borer resistance. Three genes *LTPL164*, *LTPL151*, and *LOC Os11g32100* showed more than 10-fold higher expression in 1688AI than 1688CK, suggesting their potential role in insect resistance. Collectively, our results given here provide an important foundation for further understanding of the insect resistance mechanisms of selected resistant cultivars, which could help us breed for rice resistance to *C. suppressalis*.

## Author Contributions

YW and DJ collected the samples, analyzed the data, and drafted the article. XY critically revised the article. DM selected the experimental rice cultivars. XY and XW conceived and designed the work, and approved the final version to be published.

## Conflict of Interest Statement

The authors declare that the research was conducted in the absence of any commercial or financial relationships that could be construed as a potential conflict of interest.

## Footnotes

1 http://rice.plantbiology.msu.edu/

2 http://www.genome.jp/kegg/
