# Supplementary material for: Comparative Transcriptome Analysis Between Resistant and Susceptible Rice Cultivars Responding to Striped Stem Borer (SSB), Chilo suppressalis (Walker) Infestation
Source: Front Physiol. 2018 Nov 30;9:1717. doi: 10.3389/fphys.2018.01717 (PMC6283980; doi:10.3389/fphys.2018.01717)
Supplement: Supplementary file 1 [file Table_1.pdf]

Table S1. Effect of artificialadding rice plant powder on the survival rate and larval weight of *Chilo suppressalis*

Larvae diet	Survival rate [b] (%) (mean±SD)	Weight (mg) (mean±SD)
Basal diet [a]（CK）	33.33±4.71 b	8.75±2.89 a
Basal diet+1688 powder	37.78±5.09 b	7.25±3.32 b
Basal diet+1665 powder	54.44±10.72 a	5.81±2.15 c
Basal diet+1654 powder	25.56±5.09 b	5.60±1.74 c

[a]The basal diet for rearing *C. suppressalis* was prepared according to Li et al. (2015).

[b]Larval rearing diet was prepared according to Li et al. (2015) with or without adding rice plant powder of different cultivars at the tillering period. Diet was placed in a glass tube (2cm dia., 10 cm long). Newly hatched larvae were inoculated into different diets with a density of 30 larvae / tube. After 10 days, survival rate and larvae weight were determined.
